# *Helicobacter pylori* mediated niche environment aberrations promote the progression of gastric cancer

**DOI:** 10.1016/j.gendis.2024.101207

**Published:** 2024-01-06

**Authors:** Teng Yan, Hang Sun, Yuhui Liao, Jiajian Zhou

**Affiliations:** a Nanfang Hospital, The First School of Clinical Medicine, Southern Medical University, Guangzhou, Guangdong 510515, China; b Dermatology Hospital, Southern Medical University, Guangzhou, Guangdong 510091, China

Intestinal metaplasia (IM) is a key stage in the tumorigenic Correa cascade from gastritis to final intestinal-type gastric cancer (GC).^1^ *Helicobacter pylori* (*H. pylori*) infection is the most common trigger of IM, and it promotes GC progression through induction of gastric epithelial transition.^2^ Currently, the mechanism by which *H. pylori* infection promotes tumorigenesis in IM patients is poorly understood. Zhang et al. established a single-cell transcriptomic atlas on premalignant lesions and identified biomarkers of gastric early-malignant cells, providing an opportunity to explore the molecular mechanism of GC progression at the molecular level.^3^ However, the mechanism of how *H. pylori* causes environmental aberrations in IM is poorly understood. Here, we aimed to explore the aberrations of the cellular environment associated with tumorigenesis in IM with *H. pylori* infection using single-cell RNA sequencing (scRNA-seq) analysis. Notably, we found cell type-specific immune aberrations and cell-cell contact aberrations associated with carcinogenesis in IM with *H. pylori* infection. Ultimately, we identified a key transcription factor FOXO1 which may be functional in carcinogenesis, thus providing new insights into the carcinogenic role of *H. pylori* infection in IM.

We analyzed scRNA-seq data derived from IM patients with and without *H. pylori* infection^3^ using an in-house pipeline (Table S1 and Fig. S1, 2; Supplementary materials and methods). We explored inflammatory aberrations, epithelial cell aberrations, and cell-cell contact aberrations, and attributed their contributions to environment alteration associated with tumorigenesis (Fig. S3A). Various cell lineages were identified, including mucous and secretory lineages (pit mucous cells/PMCs, gland mucous cells/GMCs), immune cells (T cells, B cells, mast cells, macrophages), stromal cells (fibroblasts, endothelial cells), stem cells, proliferative cells), and intestinal cells (goblet cells, enterocytes, enteroendocrine cells)^3^ (Fig. 1A; Fig. S3B, C, 4A). Next, we found the proportion of PMCs decreased in the *H. pylori*-infected group, while GMCs and macrophages increased (Fig. 1B). Macrophage-secreted cytokines promote the inflammation and cell growth, invasive, and metastatic behaviors of GC and correlate with the prognosis of intestinal GC (Supplementary materials and methods). When we checked the expression of inflammatory or carcinogenic cytokines, we found that macrophages had a higher expression of mediators such as IL-1B, PTGS2, and IL-8 (Fig. 1C). GMCs and PMCs are two epithelial subtypes and their aberrations indicate the process of gastric carcinogenesis (Supplementary materials and methods). Further, we found that REG1A and REG3A in GMCs and PMCs were up-regulated in the *H. pylori*-infected group, indicating that they might participate in inflammatory response stimulated by *H. pylori* infection (Fig. S4B). Further immune cell infiltration analysis demonstrated that most immune cells increased with GC, reflecting a stirred inflammatory niche (Fig. S4C). These findings supported the inflammatory role and carcinogenicity of *H. pylori* in IM.

**Figure 1:**
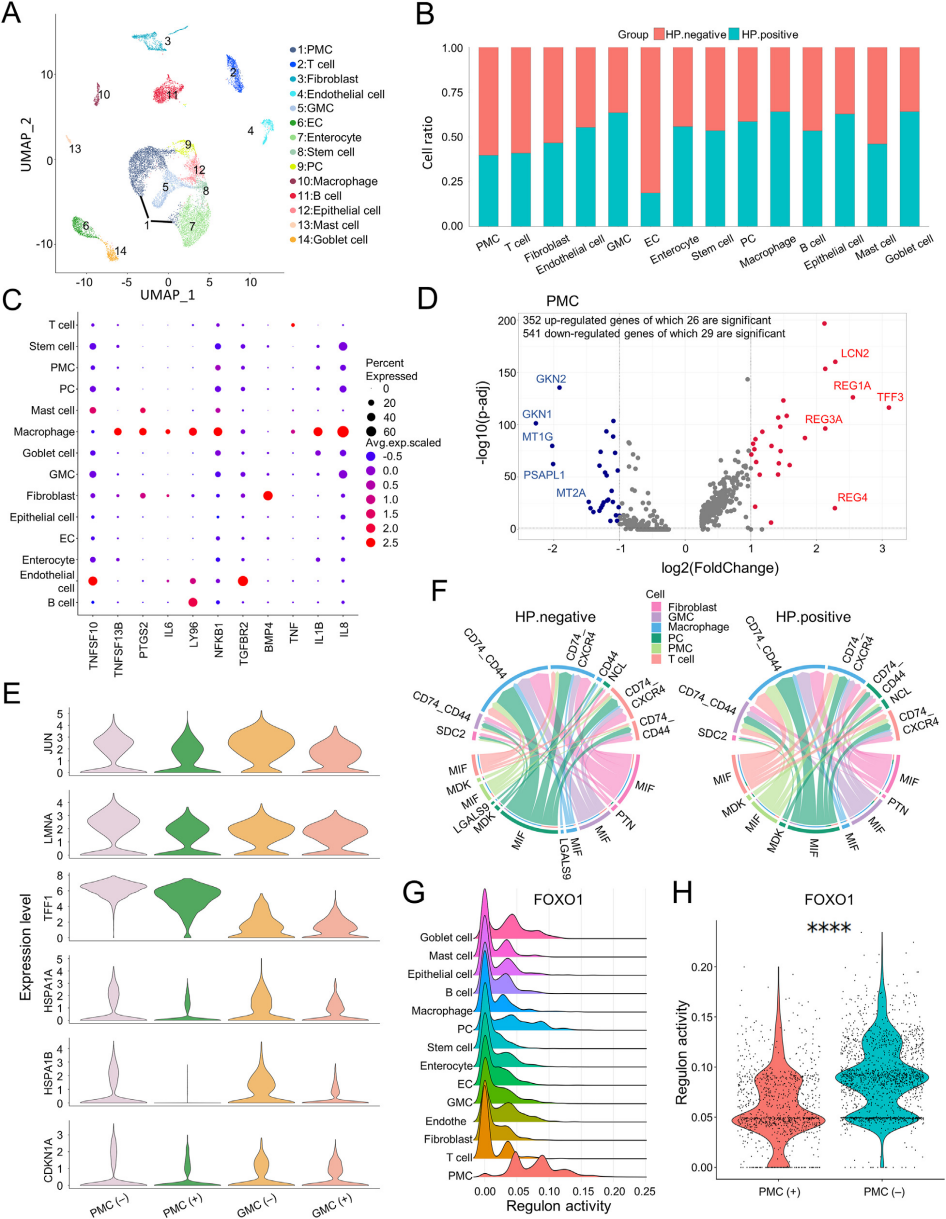
*Helicobacter pylori* (*H. pylori*)*-*mediated niche environment aberrations promote the progression of gastric cancer. **(A)** The UMAP plot showing the cell clusters in intestinal metaplasia (IM) with and without *H. pylori* infection, including PMCs (pit mucous cells), T cells, fibroblasts, endothelial cells, GMCs (antral basal gland mucous cells), ECs (enteroendocrine cells), enterocytes, stem cells, PCs (proliferative cells), macrophages, B cells, epithelial cells, mast cells, and goblet cells. **(B)** The proportion of different cell types in *H. pylori*-infected and non-infected groups. **(C)** The dot plot showing the representative genes in the NF-κB signaling pathway and inflammatory cytokines in each cell type. **(D)** The volcano plot showing the up-regulated and down-regulated genes in PMCs. Significant differential expressed genes (DEGs) were defined as log fold change ≥1 or ≤−1 and adjusted *P*-value < 0.05 using the Wilcoxon rank-sum test. **(E)** The marker genes in “negative regulation of cell population proliferation” pathways with distinct expression patterns in the *H. pylori*-infected group versus the non-infected group. **(F)** The chord plot showing the signaling pathways among PMCs, GMCs, PCs, macrophages, fibroblasts, and T cells in the *H. pylori* uninfected and infected groups. **(G)** The ridge plot showing the difference in regulon activity among different cell subtypes. The regulon activity of FOXO1 had a distinct pattern in PMCs versus other cells. **(H)** The violin plot showing the difference in regulon activity of FOXO1 in PMCs with *H. pylori* infection versus the normal sample. ^∗∗∗∗^*P* ≤ 0.0001.

*H. pylori* preferentially attaches to PMCs and influences their homeostasis to promote IM-associated GC (Supplementary materials and methods). Interestingly, differential expression genes were associated with tumorigenesis and tumor suppression in PMCs (Fig. 1D; Fig. S5A). Then, we found that the expression of JUN, LMNA, TFF1, HSPA1A, HSPA1B, and CDKN1A (enriched in “negative regulation of cell population proliferation”) are down-regulated in PMCs and GMCs with *H. pylori* infection (Fig. 1E; Fig. S6A). Further analysis showed that 143 up-regulated and 176 down-regulated genes were shared by PMCs and GMCs, indicating that PMCs and GMCs had similar responses upon *H. pylori* infection (Fig. 1D; Fig. S5B, C and Table S2). Expectedly, gene set enrichment analysis depicted a high elevation of immune response in both GMCs and PMCs, such as “antigen processing and presentation of peptide” and “humoral immune response” (Fig. S5A, D and Table S3). The heatmap and feature plot analyses also showed that the expression of some immune-related genes in these pathways had a higher expression level in GMCs and PMCs compared with other cell types (Fig. S5E, F, 6B). Our analysis demonstrated that *H. pylori* infection may promote tumorigenesis through regulating the activity of inflammatory response and inhibition of cancer-suppression function.

Next, we reconstructed a cell–cell interaction network in IM to depict the cell–cell interaction dynamics upon *H. pylori* infection. Macrophages inhibited the interactions and anti-tumor response of T cells eventually leading to the immune evasion of GC (Supplementary materials and methods). Interestingly, we found that the cell–cell contacts of PMCs and macrophages are relatively strong with *H. pylori* infection, while T cells showed a general reduction of cell–cell interaction with PMCs and macrophages (Fig. S7A, B), suggesting that the functions of macrophages and PMCs may inhibit the oncolysis of T cells. Further signaling pattern analysis showed that CXCL-mediated interactions were exclusively in the *H. pylori* negative group and VEGF-, IL1-, and BAFF-mediated interactions dominated in the *H. pylori* positive group (Fig. S7C). Subsequently, aberrations of the outgoing signal of macrophages were enriched in pathways, such as VISFATIN, BAFF, IL1, and VEGF (Fig. S7D). Macrophage migration inhibitory factor inhibits immune response and promotes inflammation response, cancer metastasis, and progression in IM and GC through binding to its receptor complexes such as CD74_CD44 and CD74_CXCR4 (Supplementary materials and methods). Expectedly, we found that the macrophage migration inhibitory factor/CD74_CD44/CD74_CXCR4 signaling pathway-mediated interactions from PMCs or T cells to macrophages were stronger in the *H. pylori* positive group, indicating aberrations of major cell-cell communications in niche environment after *H. pylori* infection (Fig. 1F; Fig. S7E).

To explore the key regulon(s) that drive the aberration of the cell-cell interaction network in GC, we established a gene regulatory network and prioritized the key regulons using SCENIC. We found that the regulons mediated by FOXO1 were specific in PMCs and the activity of FOXO1-mediated regulons was suppressed in PMCs of the *H. pylori*-infected group compared with the uninfected group, consistent with the previous finding that the FOXO1 plays a role in inhibiting the proliferation and invasion of GC cells (Fig. 1G, H; Fig. S8A, B; Supplementary materials and methods). Forkhead box (Fox) genes are a superfamily of the FOXO family that plays a function role in a wide spectrum of biological processes including tumorigenesis, metabolism, differentiation, proliferation, apoptosis, and migration^4^ (Supplementary materials and methods). Further motif screening analysis showed that the FOX family may transcriptionally regulate the expression of differential expression genes by fine-tuning their binding activities on promoter regions in PMCs (Fig. S8C).

In summary, our results showed an elevation of immune response in *H. pylori*-infected IM and dynamic changes in niche environment at the cellular and molecular levels. *H. pylori* preferentially attaches to PMCs and then leads to GKN1/2 secretion loss and premalignant inflammation (Supplementary materials and methods). Similarly, α4GnT from GMCs is a strong indicator of GC (Supplementary materials and methods). However, the mechanism of how GMCs and PMCs interact with other cells in gastric carcinogenesis is still largely unknown. Our analysis showed the dynamic of PMCs and GMCs in the inflammatory response in IM using scRNA-seq data. Interestingly, we found a decrease of the FOXO1 regulon activities in IM with *H. pylori* infection suggesting the inhibition of the tumor-suppressing process, consistent with the previous finding that FOXO1 can inhibit proliferation of GC cells.^4^ In addition, macrophage migration inhibitory factor expression is gradually increased in gastritis, IM, and GC with *H. pylori* induction, and it binds to CD74/CD44 or CD74/CXCR4 to activate macrophages to produce pro-inflammatory cytokines such as TNF-α, IFN-γ, IL-1B, IL-2, IL-6, PTGS2, and IL-8.^3^^,^^5^ Notably, other cell types may participate in GC progression, but the small sample size limits our ability to analyze the changes or pathway aberrations in rare cell types in the current study.

## Author contributions

The study design and conceptualization: J.Z., T.Y., and Y.L.; data analysis: T.Y. and H.S.; manuscript preparation, editing, and revision: T.Y. and J.Z.

## Conflict of interests

The authors declared no competing interests.

## Funding

This study is supported by the National Natural Science Foundation of China (NSFC) (32070792 to J.Z.); the Startup Foundation of Dermatology Hospital, Southern Medical University (2019RC06 to J.Z.).

## Figures and Tables

**figs1:**
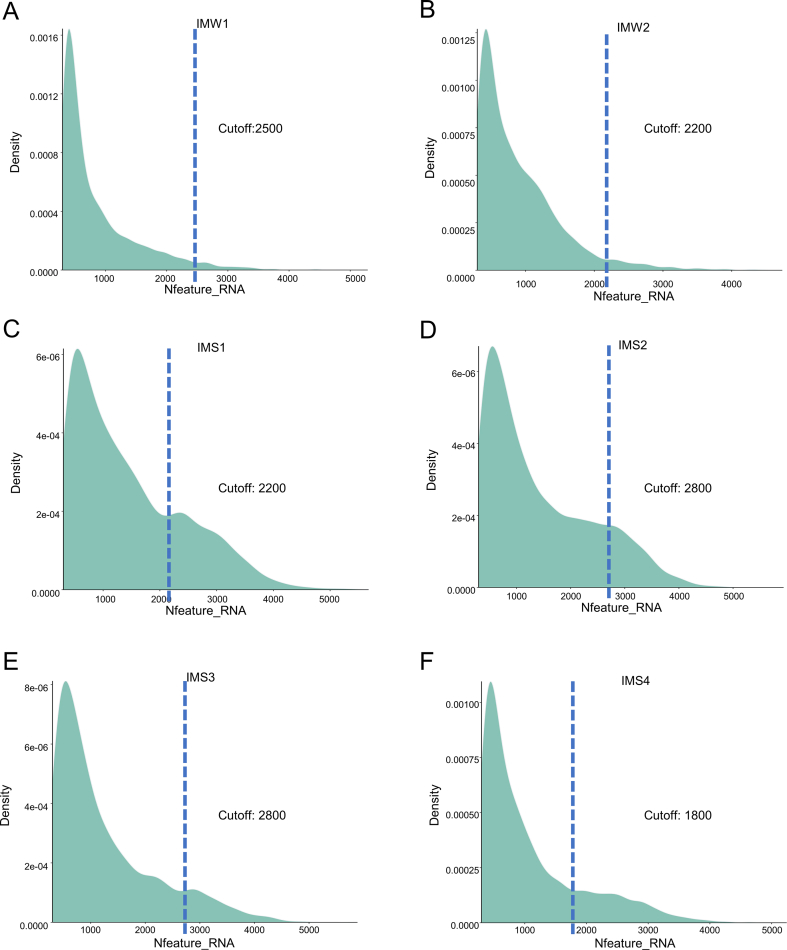
Nfeature plot shows the number of genes in each cell of all samples.

**figs2:**
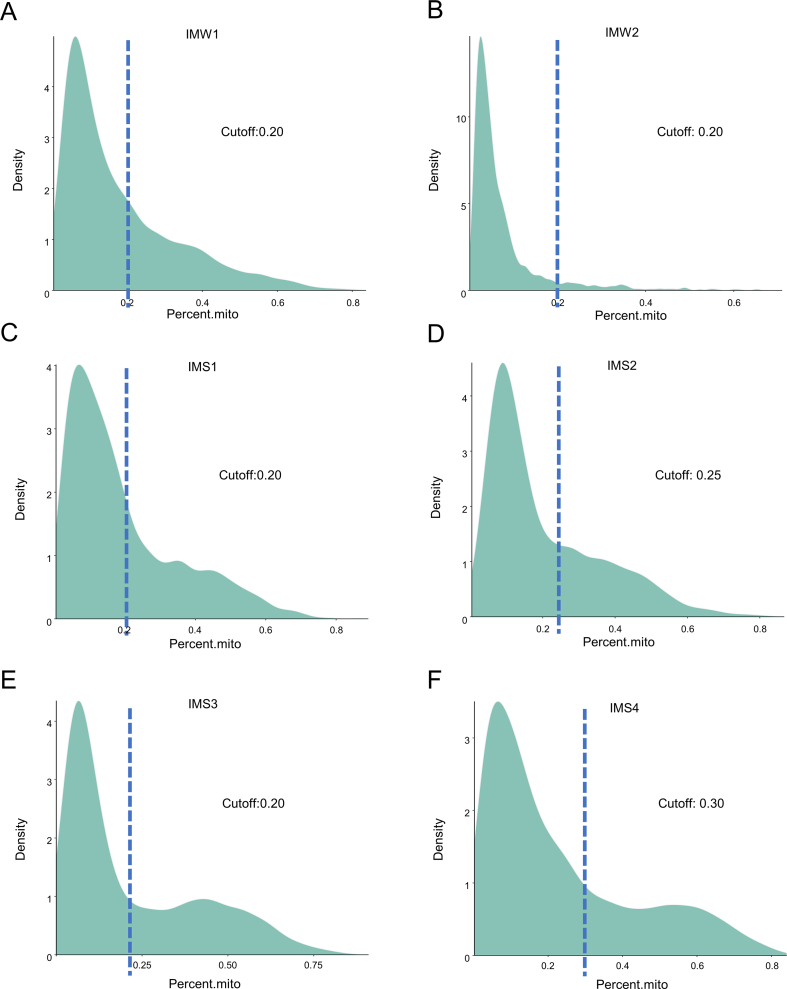
Mitoratio Plot shows the ratio of mitochondrial genes to all genes in each cell of all samples.

**figs3:**
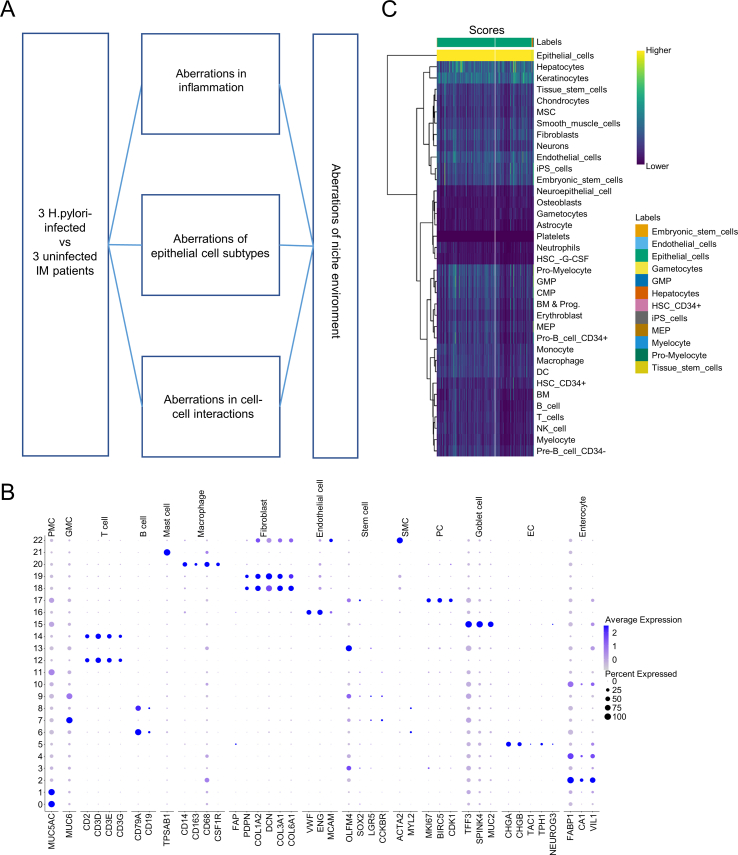
The cell-type specific marker gene sets for cell lineages in stomach. (A) The flowchart for single RNA-seq data analysis. (B) The cell-type specific markers of each cell lineages. (C) The “SingleR” analysis identifying cell lineages of the 12th cell cluster.

**figs4:**
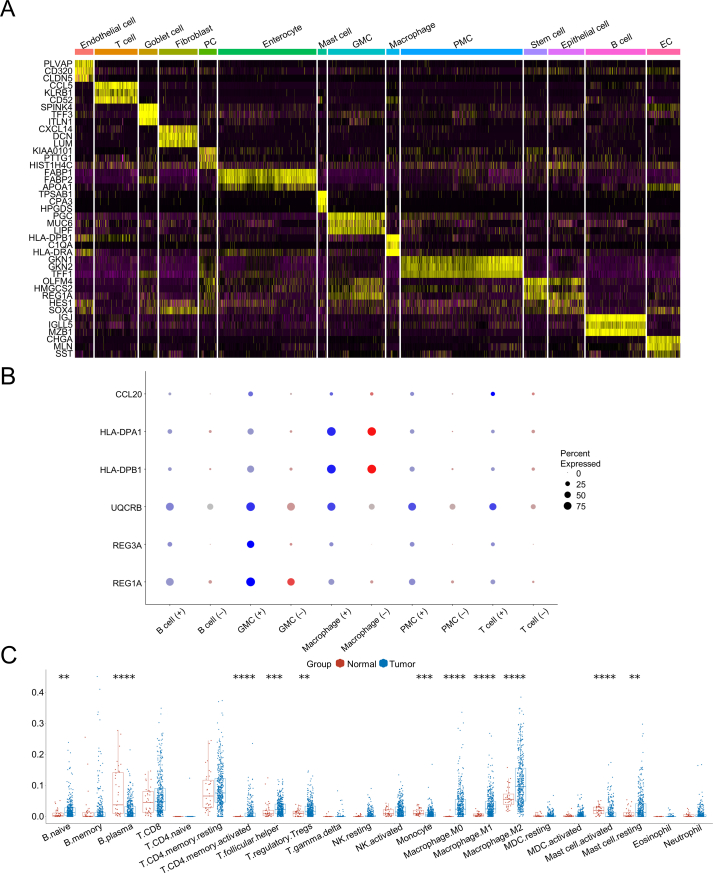
Constitute inflammation and type-specific immune aberrations in intestinal metaplasia with *H. pylori* infection. (A) Heatmap analysis of the top 3 cell-type specific markers in each cell type. (B) Dot plot showing the expressions of immune-related genes in immune and epithelial cells in *H. pylori* infected group compared with normal group. (C) Distinct immune infiltration in gastric tumor compared with normal tissue. ∗∗:p ≤ 0.01; ∗∗∗:p ≤ 0.001; ∗∗∗∗:p ≤ 0.0001.

**figs5:**
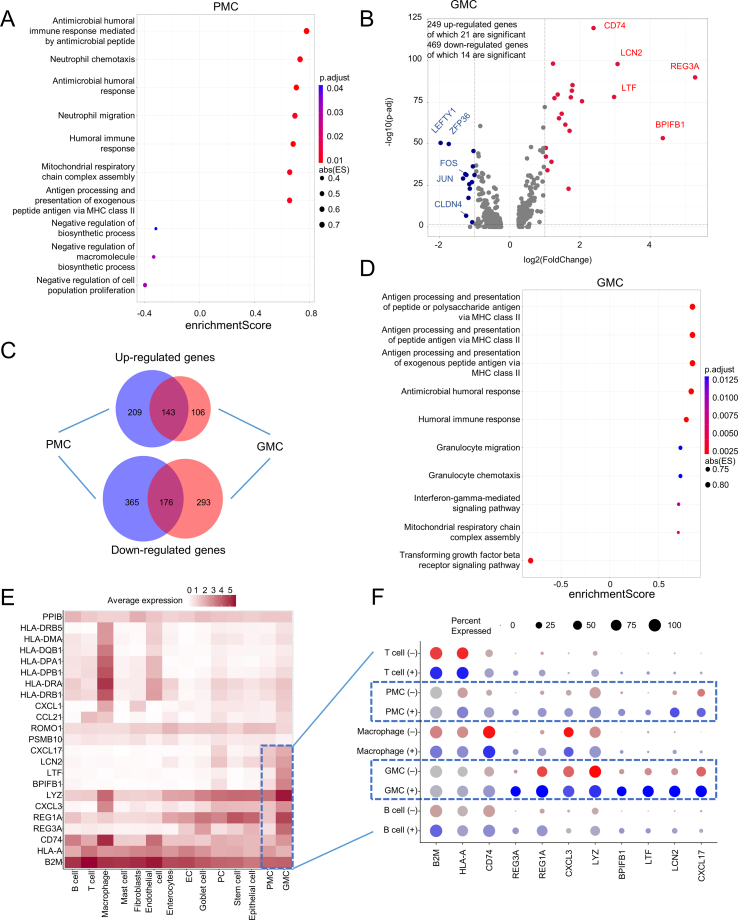
Elevation of immune-related genes in PMC and GMC indicates survival rate of gastric cancer. (A) Dot plot showing the top 7 enriched and 3 key GO Biological Process or pathways with p value<0.05 in PMC. (B) Volcano plot showing the up-regulated and down-regulated genes in GMC. Significant differential expressed genes (DEGs) are defined as log fold change ≥1 or ≤-1 and adjusted p value<0.05 using Wilcoxon rank-sum test. (C) Venn diagram showing the overlap of up-regulated and down-regulated DEGs between GMC and PMC. (D) Dot plot showing the top 10 enriched GO Biological Process or pathways with p value<0.05 in GMC. (E) Heatmap shows the expression profile of immune-related genes in the enriched pathways such as “antigen processing and presentation of peptide”, “humoral immune response” and “granulocyte migration” et al.. (F) Immune-related genes with a distinct expression pattern in PMC and GMC in *H. pylori* infected compared with non-infected group.

**figs6:**
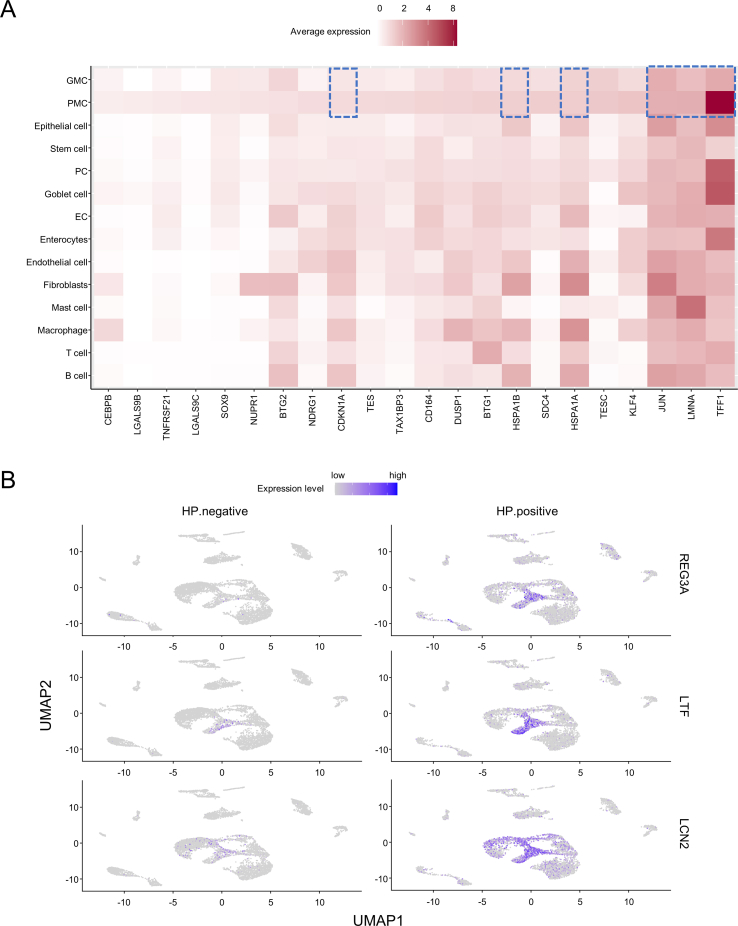
Feature plot shows a distinct expression pattern of genes in *H. pylori* infected compared with non-infected group. (A) The expression level of genes in “negative regulation of cell population proliferation” pathways ordered by avg_log2FC. (B) The distinct expression pattern of immune-related genes in *H. pylori* infected compared with non-infected group.

**figs7:**
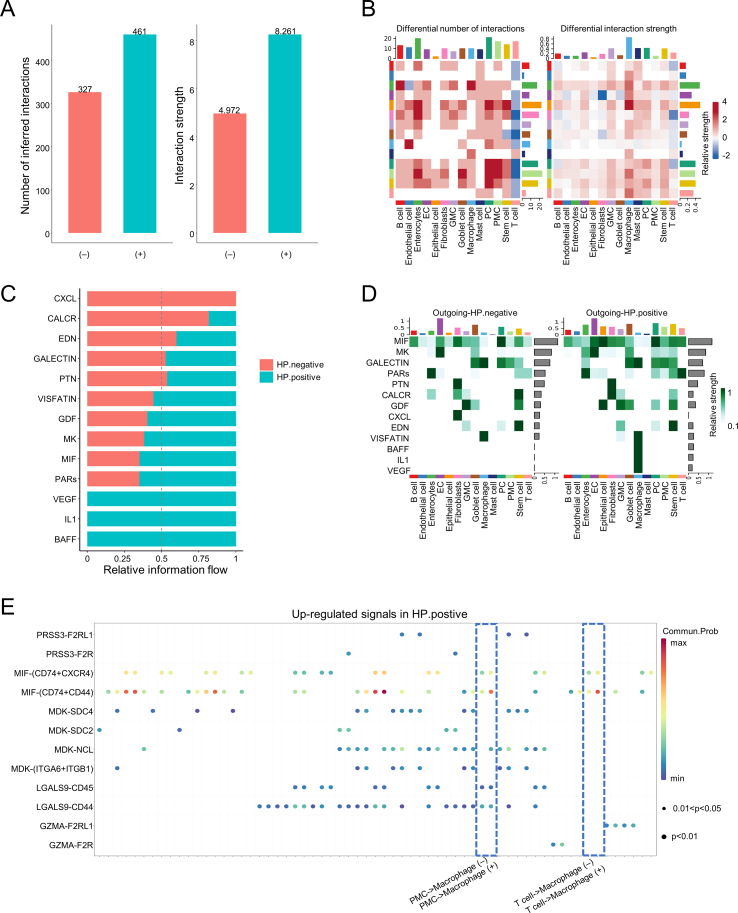
Cell-cell communications aberrations in IM patient with *H. pylori* infection. (A) Inferred interactions and interaction strength in *H. pylori* positive group compared with *H. pylori* negative group. (B) Complex heatmap shows the count (the left panel) and strength (the right panel) of cell-cell interaction among different cell types in *H. pylori* infected and non-infected group; a red block means a higher cell-cell interaction frequency or strength in *H. pylori* infected compared with non-infected group, and vice versa . (C) Signaling patterns analysis shows relative information flow strength of signaling pathways mediated interactions within two groups. (D) The outgoing signaling patterns of each cell in *H. pylori* infected and non-infected groups. (E) Dot plot shows the up-regulated signals among PMC, GMC, PC, macrophage, fibroblasts and T cell in *H. pylori* infected group.

**figs8:**
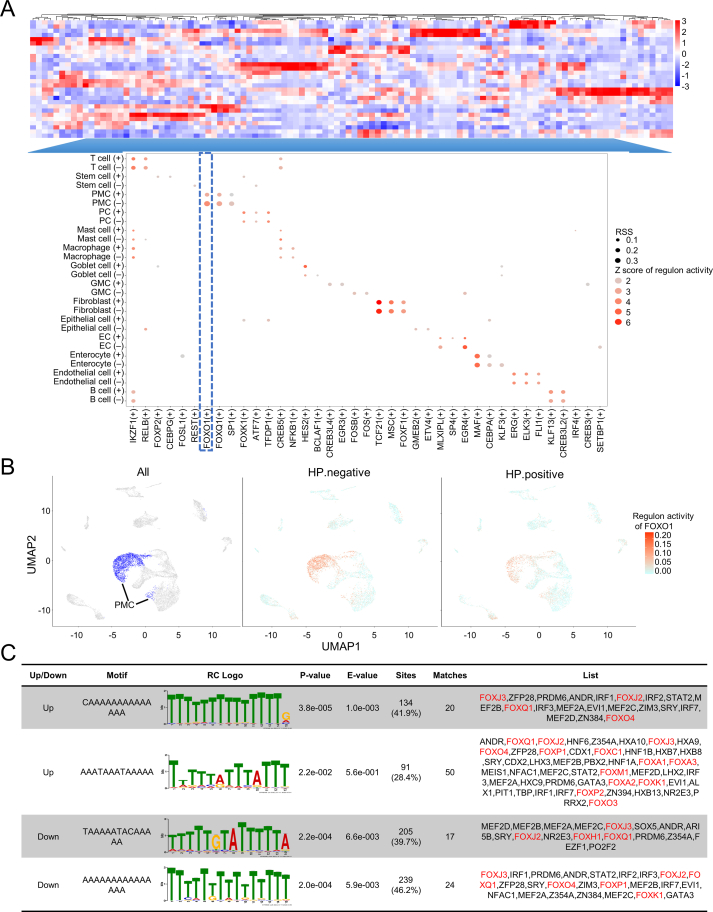
*H. pylori* infection promotes gastric cancerization through TFs regulatory network. (A) Heatmap shows the Regulon Specificity Score (RSS) of single-cell subtype-specific TFs in each cell type. (B) Feature plot shows the distribution of PMC in all cells and regulon-activity of FOXO1 in H. pylori infected compared with non-infected group. (C) Motif enrichment analysis of FOX family in proximal regions of DEGs in PMC using MEME suite.
