# Supplementary material for: Helicobacter pylori mediated niche environment aberrations promote the progression of gastric cancer
Source: Genes Dis. 2024 Jan 6;11(5):101207. doi: 10.1016/j.gendis.2024.101207 (PMC11176646; doi:10.1016/j.gendis.2024.101207)
Supplement: Multimedia component 1 [file mmc1.docx]

**Contents**

1. **Materials and Methods**

Data collection and scRNA-seq data pre-processing

Immune cell infiltration analysis in TIMER 2.0

Cell clustering analysis

Differential gene expression analysis and cell annotation

Enrichment analysis

Gene regulatory network analysis

Cell-cell contact analysis

Statistical analysis

1. **Additional analyses**

Cellular aberration of PMC and GMC in IM and their potential role in GC progression

Analysis of regulons mediated by FOXO1 using scRNA-seq

Analysis of macrophage and macrophage migration inhibitory factors (MIF)

1. **References**

**Materials and Methods**

**Data collection and scRNA-seq data pre-processing**

Six IM single-cell RNA sequencing (scRNA-seq) datasets were downloaded from Gene Expression Omnibus (GEO) databases (GSE134520)^1^ **(Table S1)**. Sex-based analysis of these six samples was not considered in this article. Because of the heterogenicity of scRNA-seq datasets, we applied a customized quality-control process for all datasets. Briefly, we examined the distributions of the number of genes and the ratio of mitochondrial genes in all cells through violin plot analysis. The number of genes at the first distinct “valley” or “flat” of the “Nfeature plot” was defined as “Max gene number” and marked by a vertical dash line to represent its cutoff value. We use this cutoff to select “the main wave” in “Nfeature plot”. **(Fig. S1).** Similarly, “Max mitoratio” was defined as the same way to keep the majority of cells in a sample, also shown as “the main wave” in “Mitoratio Plot” **(Fig. S2)**. In addition, we filtered low-quality cells with high expression of mitochondrial genes, with the number of genes < “Max gene number” and the ratio of mitochondrial genes < “Max mitoratio” in each sample **(Table S1)**. Then, we applied a similar strategy to determine the viable cells in each scRNA-seq dataset, resulting in 12861 cells for downstream analyses.

**Immune cell infiltration analysis in TIMER 2.0**

The immune cell infiltration data of gastric adenocarcinoma using the “CIBERSORT.ABS” algorithm was downloaded from TIMER 2.0 **(**[**http://timer.cistrome.org**](http://timer.cistrome.org)**)**^2–4^. The “Wilcoxon test” was used to test the difference in cell ratio between the tumor and normal group.

**Cell clustering analysis**

After data quality control, we utilized “Seurat” package (version 4.1.0) in R (version 4.0.3) for fundamental scRNA-seq data analysis. The parameters for each function were default values if not mentioned here. Data in each sample was normalized using “NormalizeData” function. Then, the high variable features were found through “FindVariableFeatures” function with parameters for “selection.method”= “vst”, “nfeatures” = 2500. The “FindIntegrationAnchors” and “IntegrateData” function with “dim” =1:50 were used to find a set of anchors between each pair of samples and integrate them with these anchors. Then “ScaleData” function was used to scale and center features in the dataset for further PCA analysis with “RunPCA” function. The first 31 principal components (PCs) and a resolution of 1.1 were set in “FindNeighbors” and “FindClusters” to cluster cell populations. The “ElbowPlot” function in “Seurat” package and “clustree” function in “clustree” package were used to determine these two parameters. Next, we used the “RunUMAP” function to construct a UMAP representation for the first 31 PCs.

**Differential gene expression analysis and cell annotation**

Cell types were annotated based on the marker gene sets of “Zhang et al.” and “SingleR” package (version 1.4.1) **(Fig. S3A, B)**^1^. Marker genes of a cluster relative to other clusters were calculated through “FindAllMarkers” function with “logfc.threshold”=1. The “FindMarkers” function was used to identify DEGs in PMC and GMC between *H. pylori* positive group and *H. pylori* negative group. Genes with log2 fold-change of the average expression between two groups > 0.25 and P < 0.05 following Bonferroni correction using “Wilcoxon rank sum test” were defined as DEGs in PMC and GMC. DEGs with absolute value of log2 fold-change > 1 were defined as significant DEGs. The “DoHeatmap”, “FeaturePlot”, “DimPlot”, “DotPlot” and “VlnPlot” functions in “Seurat” package, “ggplot2” package, “ggpubr” package and “ggsignif” package were used for visualization.

**Enrichment analysis**

Gene Set Enrichment Analysis (GSEA) is a computational method that shows distribution trends in the gene table of a predefined gene set in a phenotype-related order to reflect its contributions to phenotype^5^. The “gseGO” function in the “clusterProfiler” package was used to do GSEA on biological process (BP) of Gene Ontology (GO) terms to identify significantly upregulated and downregulated biological processes based on DEGs in PMC and GMC^6^. The top 10 enrichment pathways with a higher enrichment score and an adjusted p-value of < 0.05 in GMC were defined as “significant pathways” and plotted through bubble graphs. And 7 “significant pathways” and three pathways of interest in PMC were also visualized.

**Gene regulatory network analysis**

pySCENIC is a python implementation of the SCENIC pipeline (Single-Cell rEgulatory Network Inference and Clustering), which has a great improvement in running speed and replaces the GENIE3 module with “arboreto” package, used to infer transcription factors, calculate the activity of TFs and construct gene regulatory networks^7^. We performed pySCENIC analysis pipeline using “pyscenic” (version 0.10.0), and then performed visualization using “SCopeLoomR”, “SCENIC”, “BiocParallel”, “plotly” and “ComplexHeatmap” packages. In addition, the STREME and Tomtom in the MEME suite were used to discover potential TFs bindings in the promoter regions of the upregulated and downregulated DEGs in PMC. Motifs with p value<0.05 were selected, and the FOX family motifs were enriched in regulating upregulated DEGs and downregulated DEGs in PMC.

**Cell-cell contact analysis**

The “CellChat” R package (version 1.4.0) was used to infer the cell-cell communications for different states, further quantify the characteristics, differential interaction analysis, and visualization in the communication networks^8^. We used the “CellChat” R package to compare the cell-cell contact between two groups and quantify aberrations of exact incoming and outcoming signaling pathways to identify the primary aberrations in signaling pathways and microenvironments. Then, we extracted important cell types to visualize their interactions in multiple ligand-receptor pairs. In addition, we performed differential expression analysis in “CellChat” to identify the primary aberrations of ligand-receptor interactions with H. pylori infection and infer its role in niche environment perturbation.

**Statistical analysis**

Differences between two groups were calculated using Wilcoxon rank sum test. All statistical analyses were performed in R (v.4.0.3).

**Additional analyses**

**Cellular aberration of PMC and GMC in IM and their potential role in GC progression**

Previous studies showed aberrations of PMC and GMC have a significant role in IM associated GC. *H. pylori* preferentially attached to PMCs in its long-term colonization of the stomach, which may facilitate its further damage^9–11^. *H. pylori* caused losses of GKN1 and GKN2 in PMCs from premalignant inflammation to GC^12–14^. T. Yu et.al found that KLF4 deletion resulted in decreased PMCs and increased expression of MUC2 which is a known marker of IM and GC^15^. Similarly, a single-cell trajectory analysis found PMC is at the start point of the process to diffuse and intestinal GC patients^16^. In addition, expression of CD44 v6-containg isoforms in PMCs decreased in normal and increased in IM and GC^17^, suggesting that aberrations of PMCs may promote gastric tumorigenesis. However, *H. pylori* is always found in PMC but rarely colonizes in mucin produced by GMC, suggesting it’s not essential in colonization of *H. pylori*^18,19^. GMC produced class III mucin which had the α1,4-N-acetylglucosaminyltransferase (α4GnT) and formed GlcNAcα1→4Galβ→R^18^. The α4GnT had been found to express in tissues and peripheral blood of GC, indicating it’s a useful indicator of GC^20,21^. These results suggested the carcinogenic role of PMC and GMC aberrations.

**Analysis of regulons mediated by FOXO1 using scRNA-seq**

Forkhead box (Fox) genes are a superfamily consisted of 17 subfamilies of evolutionarily conserved transcriptional regulators, leading to tumorigenesis, metabolism, differentiation, proliferation, apoptosis, and migration^22^, which may play an important role in IM associated GC. FOXOs, a subfamily of the Fox superfamily, mediated a lot of biological processes and its dysregulation has been confirmed to influence series of cellular process such as proliferation, apoptosis, invasion, metastasis, cell cycle progression, carcinogenesis, and resistance to chemotherapeutic drugs^23^. Our results showed DEGs in PMC were universally regulated by the Fox superfamily, suggesting that *H. pylori* may affect expression profiles and cell functions in IM by regulating the upstream Fox family. Interestingly, FOXO1 inhibited proliferation and invasion of GC cell^23–25^; Mengmeng Jie et al. also found that FOXO1 is sharply downregulated in gastric cancer tissues, suggesting the inhibition of tumor-suppressing process^25^. We further found the decrease of FOXO1 in IM with *H. pylori* infection, suggesting the inhibition of tumor-suppressing response and promotion of gastric tumorigenesis by *H. pylori*. These analysis results demonstrated that the Fox family (particularly FOXO1) participates in the progression of gastric carcinogenesis.

**Analysis of macrophage and macrophage migration inhibitory factors (MIF)**

Macrophage plays an important role in the immune microenvironment of tumors, which can be further divided in to antineoplastic M1 and tumor-promoting M2 subtypes^26,27^. It secreted many cytokines like IL-10, TGF-β^28^, MMP2^29^, CXCL8^30^, COX2/MMP9^31^ and so on, which promote cell growth, invasive and metastatic behaviors of GC. Poh AR et.al found that the intestinal-type patients with a high density of M2 infiltration had a worse overall survival compared with a low density of M2 infiltration, suggesting its carcinogenic role in intestinal GC^32^. Our results also showed a higher expression of inflammatory and carcinogenic cytokines in macrophage with *H. pylori* infection Macrophage migration inhibitory factor (MIF) is a multifunctional and widely existed cytokine in the most of mammalian cells, mediating anti-pathogens immune response and promoting inflammation response, cancer metastasis and progression^33^. MIF is involved in gastric carcinogenesis because its expression in epithelial and serum tissue both gradually increased from *H. pylori*-induced gastritis, IM and GC^34^. MIF has been found to bind to an individual receptor or receptor complexes to activate its functions such as CD74/CD44, CD74/CXCR4^33,35,36^. Previous studies showed that MIF promotes inflammation after infection through activating macrophage to produce pro-inflammatory cytokines such as TNF-α, IFN -γ (Interferonγ), IL-1B, IL-2, IL-6, PTGS2 and IL-8 and the upregulated cytokines in macrophage with *H. pylori* might derive from it^1,37–39^. Notably, we also found that macrophage may inhibit the interactions and anti-tumor response of T cells in IM, eventually leading to the immune evasion of GC^40^. Additionally, MIF may trigger inflammation through interacting with the CD74/CXCR4 receptor complex and activate the PI3K/Akt signaling in T cell^36,41^. Particularly, we found that MIF-CD74_CXCR4 interaction was upregulated from T cell or PMC to macrophage in H. pylori positive group compared with *H. pylori* negative group, indicating *H. pylori* may promote the outgoing MIF signal in T cell to activate pro-inflammatory roles of macrophage via MIF-CD74_CXCR4 ligand-receptor pairs. Then, MIF can inhibit p53 directly to sustain macrophage proinflammation^42^ and inhibit the cell cycle arrest, apoptosis and promote malignant transformation^43^. In addition, MIF can also recruit CD74 and CD44 for ERK phosphorylation^44^ and further upregulation of cPLA2 and nuclear translocation of p53, thus inhibiting apoptosis and activating inflammation and malignant transformation^42,44,45^. These indicated the inflammatory and carcinogenic role of macrophage through some receptor complex like CD74_CD44 and CD74_CXCR4.

**References**

1. Zhang P, Yang M, Zhang Y, et al. Dissecting the Single-Cell Transcriptome Network Underlying Gastric Premalignant Lesions and Early Gastric Cancer. *Cell Rep*. 2019;27(6):1934-1947.e5. doi:10.1016/j.celrep.2019.04.052

2. Li T, Fu J, Zeng Z, et al. TIMER2.0 for analysis of tumor-infiltrating immune cells. *Nucleic Acids Res*. 2020;48(W1):W509-W514. doi:10.1093/nar/gkaa407

3. Li T, Fan J, Wang B, et al. TIMER: A Web Server for Comprehensive Analysis of Tumor-Infiltrating Immune Cells. *Cancer Res*. 2017;77(21):e108-e110. doi:10.1158/0008-5472.CAN-17-0307

4. Li B, Severson E, Pignon JC, et al. Comprehensive analyses of tumor immunity: implications for cancer immunotherapy. *Genome Biol*. 2016;17(1):174. doi:10.1186/s13059-016-1028-7

5. Subramanian A, Tamayo P, Mootha VK, et al. Gene set enrichment analysis: a knowledge-based approach for interpreting genome-wide expression profiles. *Proc Natl Acad Sci U S A*. 2005;102(43):15545-15550. doi:10.1073/pnas.0506580102

6. Yu G, Wang LG, Han Y, He QY. clusterProfiler: an R package for comparing biological themes among gene clusters. *Omics J Integr Biol*. 2012;16(5):284-287. doi:10.1089/omi.2011.0118

7. Van de Sande B, Flerin C, Davie K, et al. A scalable SCENIC workflow for single-cell gene regulatory network analysis. *Nat Protoc*. 2020;15(7):2247-2276. doi:10.1038/s41596-020-0336-2

8. Jin S, Guerrero-Juarez CF, Zhang L, et al. Inference and analysis of cell-cell communication using CellChat. *Nat Commun*. 2021;12(1):1088. doi:10.1038/s41467-021-21246-9

9. Boccellato F, Woelffling S, Imai-Matsushima A, et al. Polarised epithelial monolayers of the gastric mucosa reveal insights into mucosal homeostasis and defence against infection. *Gut*. 2019;68(3):400-413. doi:10.1136/gutjnl-2017-314540

10. C A, M P, M P, et al. Helicobacter pylori shows tropism to gastric differentiated pit cells dependent on urea chemotaxis. *Nat Commun*. 2022;13(1). doi:10.1038/s41467-022-33165-4

11. Hidaka E, Ota H, Hidaka H, et al. Helicobacter pylori and two ultrastructurally distinct layers of gastric mucous cell mucins in the surface mucous gel layer. *Gut*. 2001;49(4):474-480. doi:10.1136/gut.49.4.474

12. Dai J, Zhang N, Wang J, Chen M, Chen J. Gastrokine-2 is downregulated in gastric cancer and its restoration suppresses gastric tumorigenesis and cancer metastasis. *Tumour Biol J Int Soc Oncodevelopmental Biol Med*. 2014;35(5):4199-4207. doi:10.1007/s13277-013-1550-0

13. Mao W, Chen J, Peng TL, Yin XF, Chen LZ, Chen MH. Downregulation of gastrokine-1 in gastric cancer tissues and restoration of its expression induced gastric cancer cells to apoptosis. *J Exp Clin Cancer Res CR*. 2012;31(1):49. doi:10.1186/1756-9966-31-49

14. Martin TE, Powell CT, Wang Z, et al. A novel mitogenic protein that is highly expressed in cells of the gastric antrum mucosa. *Am J Physiol Gastrointest Liver Physiol*. 2003;285(2):G332-343. doi:10.1152/ajpgi.00453.2002

15. Yu T, Chen X, Lin T, et al. KLF4 deletion alters gastric cell lineage and induces MUC2 expression. *Cell Death Dis*. 2016;7(6):e2255. doi:10.1038/cddis.2016.158

16. Huang Z, Wu C, Liu X, et al. Single-Cell and Bulk RNA Sequencing Reveal Malignant Epithelial Cell Heterogeneity and Prognosis Signatures in Gastric Carcinoma. *Cells*. 2022;11(16):2550. doi:10.3390/cells11162550

17. da Cunha CB, Oliveira C, Wen X, et al. De novo expression of CD44 variants in sporadic and hereditary gastric cancer. *Lab Investig J Tech Methods Pathol*. 2010;90(11):1604-1614. doi:10.1038/labinvest.2010.155

18. Zhang MX, Nakayama J, Hidaka E, et al. Immunohistochemical demonstration of alpha1,4-N-acetylglucosaminyltransferase that forms GlcNAcalpha1,4Galbeta residues in human gastrointestinal mucosa. *J Histochem Cytochem Off J Histochem Soc*. 2001;49(5):587-596. doi:10.1177/002215540104900505

19. Nakayama J, Yeh JC, Misra AK, Ito S, Katsuyama T, Fukuda M. Expression cloning of a human alpha1, 4-N-acetylglucosaminyltransferase that forms GlcNAcalpha1-->4Galbeta-->R, a glycan specifically expressed in the gastric gland mucous cell-type mucin. *Proc Natl Acad Sci U S A*. 1999;96(16):8991-8996. doi:10.1073/pnas.96.16.8991

20. Nakajima K, Ota H, Zhang MX, et al. Expression of gastric gland mucous cell-type mucin in normal and neoplastic human tissues. *J Histochem Cytochem Off J Histochem Soc*. 2003;51(12):1689-1698. doi:10.1177/002215540305101213

21. Shimizu F, Nakayama J, Ishizone S, et al. Usefulness of the real-time reverse transcription-polymerase chain reaction assay targeted to alpha1,4-N-acetylglucosaminyltransferase for the detection of gastric cancer. *Lab Investig J Tech Methods Pathol*. 2003;83(2):187-197. doi:10.1097/01.lab.0000057001.21187.a0

22. Myatt SS, Lam EWF. The emerging roles of forkhead box (Fox) proteins in cancer. *Nat Rev Cancer*. 2007;7(11):847-859. doi:10.1038/nrc2223

23. Liu Y, Ao X, Jia Y, Li X, Wang Y, Wang J. The FOXO family of transcription factors: key molecular players in gastric cancer. *J Mol Med Berl Ger*. 2022;100(7):997-1015. doi:10.1007/s00109-022-02219-x

24. Evans-Anderson HJ, Alfieri CM, Yutzey KE. Regulation of cardiomyocyte proliferation and myocardial growth during development by FOXO transcription factors. *Circ Res*. 2008;102(6):686-694. doi:10.1161/CIRCRESAHA.107.163428

25. Jie M, Wu Y, Gao M, et al. CircMRPS35 suppresses gastric cancer progression via recruiting KAT7 to govern histone modification. *Mol Cancer*. 2020;19(1):56. doi:10.1186/s12943-020-01160-2

26. DeNardo DG, Ruffell B. Macrophages as regulators of tumour immunity and immunotherapy. *Nat Rev Immunol*. 2019;19(6):369-382. doi:10.1038/s41577-019-0127-6

27. Ngambenjawong C, Gustafson HH, Pun SH. Progress in tumor-associated macrophage (TAM)-targeted therapeutics. *Adv Drug Deliv Rev*. 2017;114:206-221. doi:10.1016/j.addr.2017.04.010

28. Mantovani A, Marchesi F, Malesci A, Laghi L, Allavena P. Tumour-associated macrophages as treatment targets in oncology. *Nat Rev Clin Oncol*. 2017;14(7):399-416. doi:10.1038/nrclinonc.2016.217

29. Wang JB, Gao YX, Ye YH, et al. CDK5RAP3 acts as a tumour suppressor in gastric cancer through the infiltration and polarization of tumour-associated macrophages. *Cancer Gene Ther*. 2023;30(1):22-37. doi:10.1038/s41417-022-00515-9

30. Piao H, Fu L, Wang Y, et al. A positive feedback loop between gastric cancer cells and tumor-associated macrophage induces malignancy progression. *J Exp Clin Cancer Res CR*. 2022;41(1):174. doi:10.1186/s13046-022-02366-6

31. Xu J, Yu Y, He X, et al. Tumor-associated macrophages induce invasion and poor prognosis in human gastric cancer in a cyclooxygenase-2/MMP9-dependent manner. *Am J Transl Res*. 2019;11(9):6040-6054.

32. Poh AR, Dwyer AR, Eissmann MF, et al. Inhibition of the SRC Kinase HCK Impairs STAT3-Dependent Gastric Tumor Growth in Mice. *Cancer Immunol Res*. 2020;8(4):428-435. doi:10.1158/2326-6066.CIR-19-0623

33. Sumaiya K, Langford D, Natarajaseenivasan K, Shanmughapriya S. Macrophage migration inhibitory factor (MIF): A multifaceted cytokine regulated by genetic and physiological strategies. *Pharmacol Ther*. 2022;233:108024. doi:10.1016/j.pharmthera.2021.108024

34. He XX, Yang J, Ding YW, Liu W, Shen QY, Xia HHX. Increased epithelial and serum expression of macrophage migration inhibitory factor (MIF) in gastric cancer: potential role of MIF in gastric carcinogenesis. *Gut*. 2006;55(6):797-802. doi:10.1136/gut.2005.078113

35. Kang I, Bucala R. The immunobiology of MIF: function, genetics and prospects for precision medicine. *Nat Rev Rheumatol*. 2019;15(7):427-437. doi:10.1038/s41584-019-0238-2

36. Bernhagen J, Krohn R, Lue H, et al. MIF is a noncognate ligand of CXC chemokine receptors in inflammatory and atherogenic cell recruitment. *Nat Med*. 2007;13(5):587-596. doi:10.1038/nm1567

37. Kamangar F, Cheng C, Abnet CC, Rabkin CS. Interleukin-1B polymorphisms and gastric cancer risk--a meta-analysis. *Cancer Epidemiol Biomark Prev Publ Am Assoc Cancer Res Cosponsored Am Soc Prev Oncol*. 2006;15(10):1920-1928. doi:10.1158/1055-9965.EPI-06-0267

38. Cheng J, Fan XM. Role of cyclooxygenase-2 in gastric cancer development and progression. *World J Gastroenterol*. 2013;19(42):7361-7368. doi:10.3748/wjg.v19.i42.7361

39. Calandra T, Roger T. Macrophage migration inhibitory factor: a regulator of innate immunity. *Nat Rev Immunol*. 2003;3(10):791-800. doi:10.1038/nri1200

40. Li J, Sun J, Zeng Z, et al. Tumour-associated macrophages in gastric cancer: From function and mechanism to application. *Clin Transl Med*. 2023;13(8):e1386. doi:10.1002/ctm2.1386

41. Schwartz V, Lue H, Kraemer S, et al. A functional heteromeric MIF receptor formed by CD74 and CXCR4. *FEBS Lett*. 2009;583(17):2749-2757. doi:10.1016/j.febslet.2009.07.058

42. Mitchell RA, Liao H, Chesney J, et al. Macrophage migration inhibitory factor (MIF) sustains macrophage proinflammatory function by inhibiting p53: regulatory role in the innate immune response. *Proc Natl Acad Sci U S A*. 2002;99(1):345-350. doi:10.1073/pnas.012511599

43. Jung H, Seong HA, Ha H. Critical role of cysteine residue 81 of macrophage migration inhibitory factor (MIF) in MIF-induced inhibition of p53 activity. *J Biol Chem*. 2008;283(29):20383-20396. doi:10.1074/jbc.M800050200

44. Shi X, Leng L, Wang T, et al. CD44 is the signaling component of the macrophage migration inhibitory factor-CD74 receptor complex. *Immunity*. 2006;25(4):595-606. doi:10.1016/j.immuni.2006.08.020

45. Mitchell RA, Metz CN, Peng T, Bucala R. Sustained mitogen-activated protein kinase (MAPK) and cytoplasmic phospholipase A2 activation by macrophage migration inhibitory factor (MIF). Regulatory role in cell proliferation and glucocorticoid action. *J Biol Chem*. 1999;274(25):18100-18106. doi:10.1074/jbc.274.25.18100
