# Supplementary material for: Helicobacter pylori mediated niche environment aberrations promote the progression of gastric cancer
Source: Genes Dis. 2024 Jan 6;11(5):101207. doi: 10.1016/j.gendis.2024.101207 (PMC11176646; doi:10.1016/j.gendis.2024.101207)
Supplement: Multimedia component 2 [file mmc2.docx]

**Table S1. The number of high-quality cells from each sample.** **IM-W, Intestinal metaplasia with wild level; IM-S, Intestinal metaplasia with severe level**

| Sample | IMW1 | IMW2 | IMS1 | IMS2 | IMS3 | IMS4 |
| --- | --- | --- | --- | --- | --- | --- |
| Sex | Male | Female | Male | Male | Male | Male |
| Cells | 1878 | 2105 | 1682 | 2506 | 1678 | 3012 |
| Max gene number | 2500 | 2200 | 2200 | 2800 | 2800 | 1800 |
| Max mitoratio | 0.20 | 0.20 | 0.20 | 0.25 | 0.20 | 0.30 |
| GSM ID | GSM3954952 | GSM3954953 | GSM3954954 | GSM3954955 | GSM3954956 | GSM3954957 |
